# Power of the 2-locus TDT for testing the interaction of two susceptibility genes

**DOI:** 10.1186/1753-6561-1-s1-s65

**Published:** 2007-12-18

**Authors:** Salma Kotti, Mathieu Bourgey, Françoise Clerget-Darpoux

**Affiliations:** 1 Université Paris-Sud, UMR-S535, 94817 Villejuif, France; 2 Inserm U535, Hôpital Paul Brousse, BP1000, 94817 Villejuif, France

## Abstract

We recently proposed a new strategy: 2-locus TDT for detecting two susceptibility genes through their interaction in trio families. We apply our method to two candidate genes, A and C, on the Genetic Analysis Workshop 15 (GAW15) simulated rheumatoid arthritis data and study the power to identify an interactive effect of these genes.

This study was performed with full knowledge of the answers.

## Introduction

Most multifactorial diseases result from complex interactions among multiple genes with modest effects and various environmental factors. Current strategies usually aim to detect the individual effect of each factor.

We have recently proposed a new strategy: the 2-locus TDT (transmission-disequilibrium test), which tests for interaction between two candidate genes [1]. Like the one-locus TDT [2], it applies to trio families including the patient and his two parents. Our method is used in the context of studying good candidate pathways. Indeed, we assume prior information that the disease under study is probably caused by particular genes recognized as key elements of the metabolic pathway. We apply the 2-locus TDT to Problem 3 simulated Genetic Analysis Workshop 15 (GAW15) rheumatoid arthritis (RA) data and compute its power for detecting the interaction of two susceptibility genes.

## Data

The GAW15 Problem 3 consisted of 100 replicates of 1500 nuclear families with two affected sibs. From each replicate, we extracted 1500 trio families: one affected (the index case) and his two parents.

We focus the analyses on two candidate genes in the RA susceptibility: Locus A on chromosome 16 and Locus C on chromosome 6. Locus A has an indirect effect on RA hazard. Its implication on the susceptibility to the disease depends on the HLA-DRB1 locus located on chromosome 6. Locus C is in strong linkage disequilibrium (LD) with HLA-DRB1. Both genes are involved directly or indirectly in the RA outcome through the HLA-DRB1 gene and thus do not act independently. We apply the 2-locus TDT to the SNPs indicated as functional in the two candidate genes in each replicate of 1500 trios.

## Notation

We denote A_1_, A_2 _and C_1_, C_2 _the SNP alleles of genes A and C, respectively. For each patient, there are nine possible genotypes that we denote A_i_A_j_C_k_C_l_, where A_i_, A_j _and C_k_, C_l _are the alleles inherited from the two parents at the loci A and C, respectively. Subscripts i, j, k and l can be either 1 or 2. A_2 _and C_2 _are considered to be the high risk alleles and A_2_A_2_C_2_C_2 _are the reference genotype.

We denote the nine penetrances P(affected | A_i_A_j_C_k_C_l_) by *f*_*ijkl *_and by *ε*_*ijkl *_the eight penetrances relatively to the reference genotype A_2_A_2_C_2_C_2_. Thus, *ε*_2222 _= 1. Let M_G _be the general model in which *ε*_*ijkl *_may take any value between 0 and 1. When the two genes A and B act independently, the lines and the columns of the relative penetrance matrix are proportional (multiplicative matrix) and may be written with four parameters: μ, ν, ϕ and π (see Table 1). The corresponding restricted model is denoted M_R_.

**Table 1:** Penetrance estimation under the restricted model

	Gene A		
	
Gene C	A_1_A_1_	A_1_A_2_	A_2_A_2_
C_1_C_1_	ϕμ	πμ	μ
C_1_C_2_	ϕν	πν	ν
C_2_C_2_	ϕ	π	1

## Method

The 2-locus TDT is achieved in two steps. In the first step, the parameters (μ, ν, ϕ, and π) of the restricted model and *ε*_*ijkl *_of the general model are estimated by their maximum likelihood estimator (MLE). In the second one, the restricted model is compared to the general one by a maximum likelihood ratio test.

The estimation of these parameters requires specifying the allele frequencies at each locus. This was achieved by the affected family-based controls (AFBAC) method, using the parental alleles untransmitted to the patients from which the family was ascertained [3]. Here, the families were not ascertained through an affected patient but through a patient with an affected sib. Our parameter estimation must also take into account this specific mode of ascertainment and consequently was achieved by using the marker association segregation chi-square (MASC) program [4].

We denote by max_G_L_G _and max_R_L_R _the likelihood values for the estimated parameters of the general and restricted models, respectively. The likelihood ratio test (LRT) was calculated as follows:

$$LRT=-2\ln\frac{\max_{R}L_{R}}{\max_{G}L_{G}}.$$

This ratio follows, under the null hypothesis, a chi-square distribution with four degrees of freedom (df) corresponding to the difference in the number of parameters between the two models.

For each replicate of 1500 trios, the LRT was computed and the significance of the test was assessed in order to evaluate the power of our test in the present situation.

## Results

**Figure 1:**
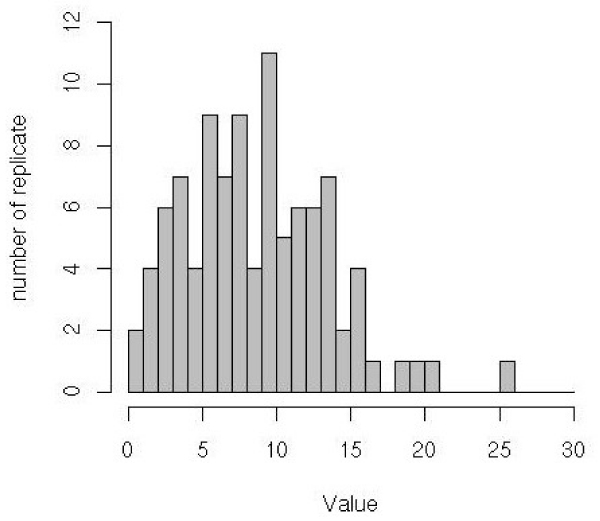
Distribution of the LRT values.

**Figure 2:**
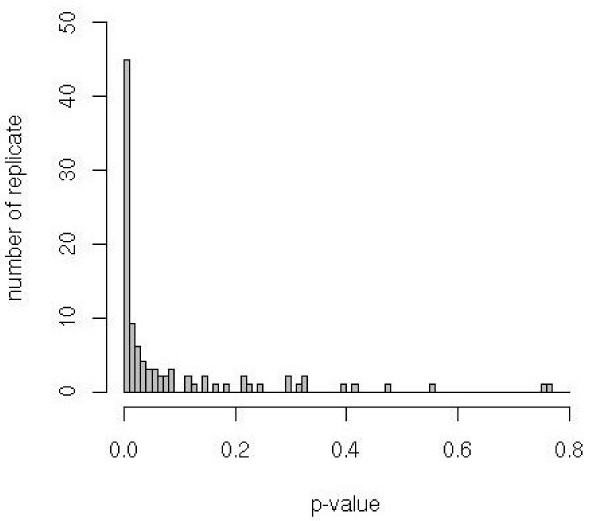
Distribution of the LRT *p*-values.

Because patients with genotypes A_1_A_1_C_1_C_1_, A_1_A_2_C_1_C_1_, and A_2_A_2_C_1_C_1 _are very rare in all replicates, we fixed the relative penetrances of these genotypes at 10^-3^. Consequently, under the general model, we estimate only five parameters and, under the restricted model, only three parameters ν, ϕ, and π. Thus, in the present situation, we have 2 degrees of freedom for the LRT. Figures 1 and 2 show the distributions of the LRT and *p*-values in 98 replicates (the program failed in 2 replicates), respectively. There is a good percentage of replicates (44/98) for which the test was significant at 1% level. The power of 2-locus TDT test (at a 5% level) is 66%.

We illustrate the variation of the LRT and *p*-values on two contrasting replicates: 9 and 27. In the Replicate 27, there is a strong evidence for interaction: LRT = 14.35 and *p *= 7 × 10^-4^. In contrast, we did not find interaction in Replicate 9 because the LRT = 2.49 and *p *= 0.29.

Tables 2 and 3 show the parameter estimates under the general model for Replicates 9 and 27, respectively. The parameter estimates under the restricted model are ν = 0.135, ϕ = 0.688, and π = 0.886 for Replicate 9 and ν = 0.168, ϕ = 0.682, and π = 0.909 for replicate 27. It is clear that the departure from multiplicativity in the relative penetrance matrix of the general model is stronger for Replicate 27 than for Replicate 9. In particular, for Replicate 27, the relative penetrance for the genotype A_1_A_2_C_2_C_2 _is half of the one expected under the restricted model.

**Table 2:** Replicate 9: Matrix of relative penetrances for the general model

	Gene A		
	
Gene C	A_1_A_1_	A_1_A_2_	A_2_A_2_
C_1_C_1_	0.001	0.001	0.001
C_1_C_2_	**0.105**^a^	**0.109**	**0.113**
C_2_C_2_	**0.668**	**0.879**	1

^a ^Bold indicates parameter estimates.

**Table 3:** Replicate 27: Matrix of relative penetrances for the general model

	Gene A		
	
Gene C	A_1_A_1_	A_1_A_2_	A_2_A_2_
C_1_C_1_	0.001	0.001	0.001
C_1_C_2_	**0.137**	**0.133**	**0.083**
C_2_C_2_	**0.623**	**0.868**	1

^a ^Bold indicates parameter estimates.

## Discussion

In this paper, we studied the performance of the 2-locus TDT for detecting the interaction of the genes A and C in the susceptibility to RA simulated data and show that interaction may be detected with a power of 66% at a 5% level.

The 2-locus TDT searches for a departure from multiplicativity in the two-locus penetrance matrix. This departure cannot be observed without an effect of the two genes. Therefore, the 2-locus TDT may also be used for detecting the effect of two candidate genes through their interaction.

More generally, the 2-locus TDT may be particularly interesting in the situation of weak effect of one or both genes and strong interaction. In such a situation, traditional methods will fail because they will stop in the absence of noticeable main effect. In fact, they ignore the possibility that the interactive effect of multilocus functional genetic variants may play a larger role than the single-locus effect in the determinism of one trait [1].

Our test applies to candidate genes already recognized as key elements of a metabolic pathway thought to be involved in the disease pathogenesis. There are difficulties in good candidate choice and the use of tagSNPs as markers for most genetic variations in the human genome has become a cornerstone of genetic research. There are, however, reasons to think that the expectations they have raised are somewhat inflated and may lead to unwarranted neglect of other strategies. In particular, we believe in particular that identification of biologically relevant pathways followed by candidate gene strategies may be quite effective at identifying new variants and their interaction in disease susceptibility [5].

## Competing interests

The author(s) declare that they have no competing interests.
